# Taxonomy and phylogeny of *Lopharia* s.s., *Dendrodontia*, *Dentocorticium* and *Fuscocerrena* (Basidiomycota, Polyporales)

**DOI:** 10.3897/mycokeys.32.23641

**Published:** 2018-03-15

**Authors:** Shi-Liang Liu, Karen K. Nakasone, Sheng-Hua Wu, Shuang-Hui He, Yu-Cheng Dai

**Affiliations:** 1 Institute of Microbiology, Beijing Forestry University, Beijing 100083, China; 2 Center for Forest Mycology Research, Northern Research Station, U.S. Forest Service, One Gifford Pinchot Drive, Madison, WI 53726-2398, USA; 3 Department of Biology, National Museum of Natural Science, Taichung 40419, Taiwan; 4 Beijing Advanced Innovation Centre for Tree Breeding by Molecular Design, Beijing Forestry University, Beijing 100083, China

**Keywords:** Corticioid fungi, dendrohyphidia, species complex, wood-inhabiting fungi

## Abstract

Eleven taxa of *Lopharia* s.s., *Dendrodontia*, *Dentocorticium* and *Fuscocerrena* in Polyporales are included in the phylogenetic analyses of nuc rDNA ITS1-5.8S-ITS2 (ITS), D1-D2 domains of nuc 28S rDNA (28S) and RNA polymerase II second-largest subunit (*rpb2*) sequences. New species *Lopharia resupinata* and *L. sinensis* are described and illustrated. *Lopharia resupinata*, from south-eastern China, is closely related to *L. ayresii*, and *L. sinensis*, from northern China, is related to *L. cinerascens* and *L. mirabilis*. *Lopharia mirabilis* specimens from temperate to tropical areas with varied hymenophore configurations all cluster together in a fully supported clade. *Dendrodontia* and *Fuscocerrena* are shown to be synonyms of *Dentocorticium*, which is phylogenetically related to *Lopharia*. Four new combinations, *Dentocorticium bicolor*, *D. hyphopaxillosum*, *D. portoricense* and *D. taiwanianum*, are proposed. Revised generic descriptions of *Lopharia* and *Dentocorticium* are provided with keys to the six accepted species in each genus. A list of all names in *Lopharia* and *Dentocorticium* are presented with their current taxonomic status. Type specimens of *Dentocorticium brasiliense* and *D. irregulare* were examined and determined to be later synonyms of *Punctularia subhepatica* and *Diplomitoporus daedaleiformis*, respectively.

## Introduction

The genus *Lopharia* s.s., typified by *L. lirellosa* Kalchbr. & MacOwan (= *Radulum mirabile* Berk. & Broome), is characterised by a dimitic hyphal system with clamped generative hyphae, large basidia and basidiospores and large, encrusted, hyaline, thick-walled cystidia (Hjortstam and Ryvarden 1990, Boidin and Gilles 2002, Bernicchia and Gorjón 2010). Of 35 taxa placed in *Lopharia*, Hjortstam and Ryvarden (1990) accepted only *L. cinerascens* (Schwein.) G. Cunn. and *L. mirabilis* (Berk. & Broome) Pat. and Boidin and Gilles (2002) additionally accepted *L. pseudocinerascens* Boidin & Gilles. Welden (1975, 2010) adopted a broad interpretation of *Lopharia* that included species of *Porostereum* Pilát. A few phylogenetic studies that have included *Lopharia* s.s. and *Porostereum spadiceum* (Pers.) Hjortstam & Ryvarden (generic type) showed that they are distantly related (Ko et al. 2001, Yoon et al. 2003, Wu et al. 2007, Jang et al. 2016). Both genera are included in the Polyporales with *Lopharia* in the Polyporaceae and *Porostereum* in the Phanerochaetaceae (Justo et al. 2017).

*Dentocorticium* (Parmasto) M.J. Larsen & Gilb. was segregated from *Laeticorticium* Donk to accommodate *L. ussuricum* Parmasto (generic type) and *Hydnum sulphurellum* Peck (Larsen and Gilbertson 1974) because they lack probasidia. Subsequently, nine species were described or transferred to the genus (Larsen and Gilbertson 1977, Ryvarden 1978, Domański 1988, Boidin et al. 1996, Boidin and Gilles 1998, Duhem and Michel 2009).

*Dendrodontia* Hjortstam & Ryvarden (generic type *Grandinia bicolor* P.H.B. Talbot) is similar to *Dentocorticium* in possessing tuberculate to odontoid hymenophore, dendrohyphidia and thin-walled smooth basidiospores, but differs by its dimitic hyphal system with brown skeletal hyphae (Hjortstam and Ryvarden 1980, Boidin and Gilles 1998). The monotypic genus *Fuscocerrena* Ryvarden was erected for *Polyporus portoricensis* Spreng. ex Fr. This taxon is characterised by dark brown, effused, effused-reflexed to pileate basidiocarps with a poroid to spinose hymenophore, a dimitic hyphal system with brown skeletal hyphae and dendrohyphidia (Ryvarden 1982). Except for the variable hymenophore configuration and greenish-yellow hymenial surface, *F. portoricensis* (Spreng. ex Fr.) Ryvarden is similar to many species of *Dendrodontia* and *Dentocorticium* at the microscopic level.

Morphologically, *Lopharia* s.s. is distinct from *Dentocorticium* and *Dendrodontia* but are phylogenetically closely related as shown in phylogenetic studies based on two to six taxa (Yoon et al. 2003, Wu et al. 2007, Justo and Hibbett 2011, 2017, Jang et al. 2016). In this study, eleven taxa of *Lopharia* s.s., *Dentocorticium*, *Dendrodontia* and *Fuscocerrena* from North America and East Asia were included in phylogenetic analyses of a concatenated 3-gene dataset of ITS, 28S and *rpb2* sequences.

## Materials and methods

**Morphological studies.** Voucher specimens are deposited in the herbarium of Beijing Forestry University (BJFC), the National Museum of Natural Science in Taiwan (TNM) and the Centre for Forest Mycology Research (CFMR). Samples for microscopic examination were mounted in 0.2 % cotton blue in lactic acid, 1 % phloxine and Melzer’s reagent. The following abbreviations are used: L = mean spore length, W = mean spore width, Q = L/W ratio, n (a/b) = number of spores (a) measured from given number of specimens (b). Colour codes and names follow Kornerup and Wanscher (1978).

**DNA extraction and sequencing.** A CTAB plant genome rapid extraction kit-DN14 (Aidlab Biotechnologies Co. Ltd, Beijing) was employed for DNA extraction and PCR amplification from dried specimens. The ITS, 28S and *rpb2* gene regions were amplified with the primer pairs ITS5 and ITS4 (White et al. 1990), LR0R and LR7 (http://www.biology.duke.edu/fungi/mycolab/primers.htm) and *rpb2*-f5F and *rpb2*-7.1R (Liu et al. 1999, Matheny et al. 2007), respectively. The PCR procedures for ITS and 28S followed Liu et al. (2017), while the procedure for *rpb2* was the same as Justo and Hibbett (2011). DNA sequencing was performed at Beijing Genomics Institute and the sequences are deposited in GenBank (Table 1).

**Table 1.** Species and sequences used in the phylogenetic analyses. Newly generated sequences are set in bold.

Taxa	Voucher	Locality	ITS	28S	rpb2
*Amauroderma rugosum*	ML 56	Japan	AB509712	AB368061	AB368119
*Boletopsis leucomelaena*	AFTOL 1527	USA	DQ484064	DQ154112	GU187820
*Climacodon septentrionalis*	AFTOL 767	USA	AY854082	AY684165	AY780941
*Coriolopsis gallica*	RLG-7630-Sp	USA	JN165013	JN164814	JN164821
*Coriolopsis trogii*	RLG-4826-Sp	USA	JN164993	JN164808	JN164867
*Daedaleopsis confragosa*	WD 747	Japan	GU731549	AB368062	AB368120
*Datronia mollis*	RLG-6304-Sp	USA	JN165002	JN164791	JN164872
*Datronia scutellata*	RLG-9584-T	USA	JN165004	JN164792	JN164873
***Dendrocorticium bicolor***	**He 2772**	**China**	**MF626354**	**MF626378**	–
***Dendrocorticium bicolor***	**He 2757**	**China**	**MF626355**	**MF626379**	–
***Dendrocorticium portoricense***	**He 2161**	**USA**	**MF626356**	**MF626380**	**MF626397**
***Dendrocorticium portoricense***	**He 2202**	**USA**	**MF626357**	**MF626381**	–
***Dendrocorticium taiwanianum***	**He 3383**	**China**	**MF626361**	**MF626385**	–
***Dendrocorticium taiwanianum***	**He 4615**	**China**	**MF626362**	**MF626386**	–
***Dendrocorticium taiwanianum***	**He 3777**	**China**	–	**MF626388**	–
***Dendrocorticium taiwanianum***	**Wu 9907-1 (type)**	**China**	**MF626363**	**MF626387**	–
***Dendrocorticium ussuricum***	**He 3322**	**China**	**MF626360**	**MF626384**	**MF626399**
***Dendrocorticium ussuricum***	**He 3278**	**China**	**MF626358**	**MF626382**	–
***Dendrocorticium ussuricum***	**He 3294**	**China**	**MF626359**	**MF626383**	**MF626398**
*Dentocorticium sulphurellum*	T 609	Canada	JN165015	JN164815	JN164875
*Earliella scabrosa*	PR 1209	Puerto Rico	JN165009	JN164793	JN164866
*Fomitopsis pinicola*	AFTOL 770	USA	AY854083	AY684164	AY786056
*Ganoderma lucidum*	WD 565	Japan	EU021460	AB368068	AB368126
*Ganoderma tsugae*	AFTOL 771	USA	DQ206985	AY684163	DQ408116
*Grifola sordulenta*	AFTOL 562	USA	AY854085	AY645050	AY786058
*Hydnellum geogenium*	AFTOL 680	USA	DQ218304	AY631900	DQ408133
*Irpex lacteus*	TM 03-480	Japan	AB079264	EU522839	DQ408117
*Lentinus squarrosulus*	WD 1729	Japan	GU001951	AB368071	AB368129
*Lentinus tigrinus*	MUCL 22821	Japan	AF516520	AB368072	AB368130
*Lenzites betulinus*	AJ 150	USA	JN164915	–	–
***Lopharia ayresii***	**He 20120724-4**	**China**	**MF626352**	**MF626375**	–
***Lopharia ayresii***	**He 2778**	**China**	**MF626353**	**MF626376**	–
***Lopharia cinerascens***	**He 2188**	**USA**	**MF626350**	**MF626373**	**MF626395**
***Lopharia cinerascens***	**He 2228**	**USA**	**MF626351**	**MF626374**	–
***Lopharia resupinata***	**He 4401 (type)**	**China**	–	**MF626377**	**MF626396**
***Lopharia mirabilis***	**Dai 5147**	**China**	**MF626342**	**MF626365**	**MF626389**
***Lopharia mirabilis***	**Yuan 2532**	**China**	**MF626343**	**MF626366**	**MF626390**
***Lopharia mirabilis***	**Dai 5598**	**China**	**MF626341**	**MF626364**	–
***Lopharia mirabilis***	**He 4558**	**China**	**MF626344**	**MF626367**	–
***Lopharia mirabilis***	**Dai 14978**	**China**	**MF626345**	**MF626368**	**MF626391**
***Lopharia mirabilis***	**Dai 13722**	**China**	**MF626346**	**MF626369**	**MF626392**
***Lopharia sinensis***	**He 2428 (type)**	**China**	**MF626347**	**MF626370**	**MF626393**
***Lopharia sinensis***	**He 2510**	**China**	**MF626348**	**MF626371**	**MF626394**
***Lopharia sinensis***	**He 2424**	**China**	**MF626349**	**MF626372**	–
*Lopharia* sp.	FP-105043	USA	JN165019	JN164813	JN164874
*Phanerochaete chrysosporium*	FPL 5175	USA	AF854086	AF287883	–
*Phlebia radiata*	FPL 6140	USA	AY854087	AF287885	AY218502
*Polyporus squamosus*	AFTOL 704	USA	DQ267123	AY629320	DQ408120
*Polyporus umbellatus*	WD 719	Japan	EU442276	AB368109	AB368166
*Pseudofavolus cucullatus*	WD 2157	Japan	AF516601	AB368114	AB368170
*Pycnoporus sanguineus*	PR-SC-95	Puerto Rico	JN164982	JN164795	JN164858
*Pycnoporus cinnabarinus*	ZW 02-30	China	DQ411525	AY684160	DQ408121
*Trametes ectypa*	FP-106037-T	USA	JN164929	JN164803	JN164848
*Trametes hirsuta*	RLG-5133-T	USA	JN164941	JN164801	JN164854
*Trametes versicolor*	FP-135156-Sp	USA	JN164919	JN164809	JN164850
*Trametopsis cervina*	TJV-93-216-Sp	USA	JN165020	JN164796	JN164877

**Phylogenetic analyses.** The molecular phylogeny used a combined dataset of ITS, 28S and *rpb2* sequences. Justo and Hibbett (2011) was consulted for taxon sampling and outgroup selection. The sequences were aligned using the MAFFT v.6 (Katoh and Toh 2008, http://mafft.cbrc.jp/alignment/server/). Alignments were optimised manually in BioEdit 7.0.5.3 (Hall 1999) and deposited at TreeBase (http://treebase.org/treebase-web/home.html, submission ID: 21717).

Maximum Likelihood (ML), Maximum Parsimony (MP) and Bayesian Inference (BI) analyses were performed by using RAxML 7.2.6 (Stamatakis 2006), PAUP* 4.0b10 (Swofford 2002) and MrBayes 3.1.2 (Ronquist and Huelsenbeck 2003), respectively. In ML analysis, statistical support values were obtained from rapid bootstrapping of 1000 replicates using default settings for other parameters. In MP analysis, gaps in the alignments were treated as missing data. Trees were generated using 100 replicates of random stepwise addition of sequence and tree-bisection reconnection (TBR) branch-swapping algorithm with all characters given equal weight. Branch supports for all parsimony analyses were estimated by performing 1000 bootstrap replicates (Felsenstein 1985) with a heuristic search of 10 random-addition replicates for each bootstrap replicate. For BI, best models of evolution were estimated by using MrModeltest 2.2 (Nylander 2004) and the Bayesian posterior probabilities (BPP) were determined by Markov Chain Monte Carlo sampling in MrBayes 3.1.2. Four simultaneous Markov chains were run for two million generations and trees were sampled every 100th generation. The first quarter of the trees, which represented the burn-in phase of the analyses, were discarded and the remaining trees were used to calculate posterior probabilities in the majority rule consensus tree.

### Phylogeny results

**Figure 1.**
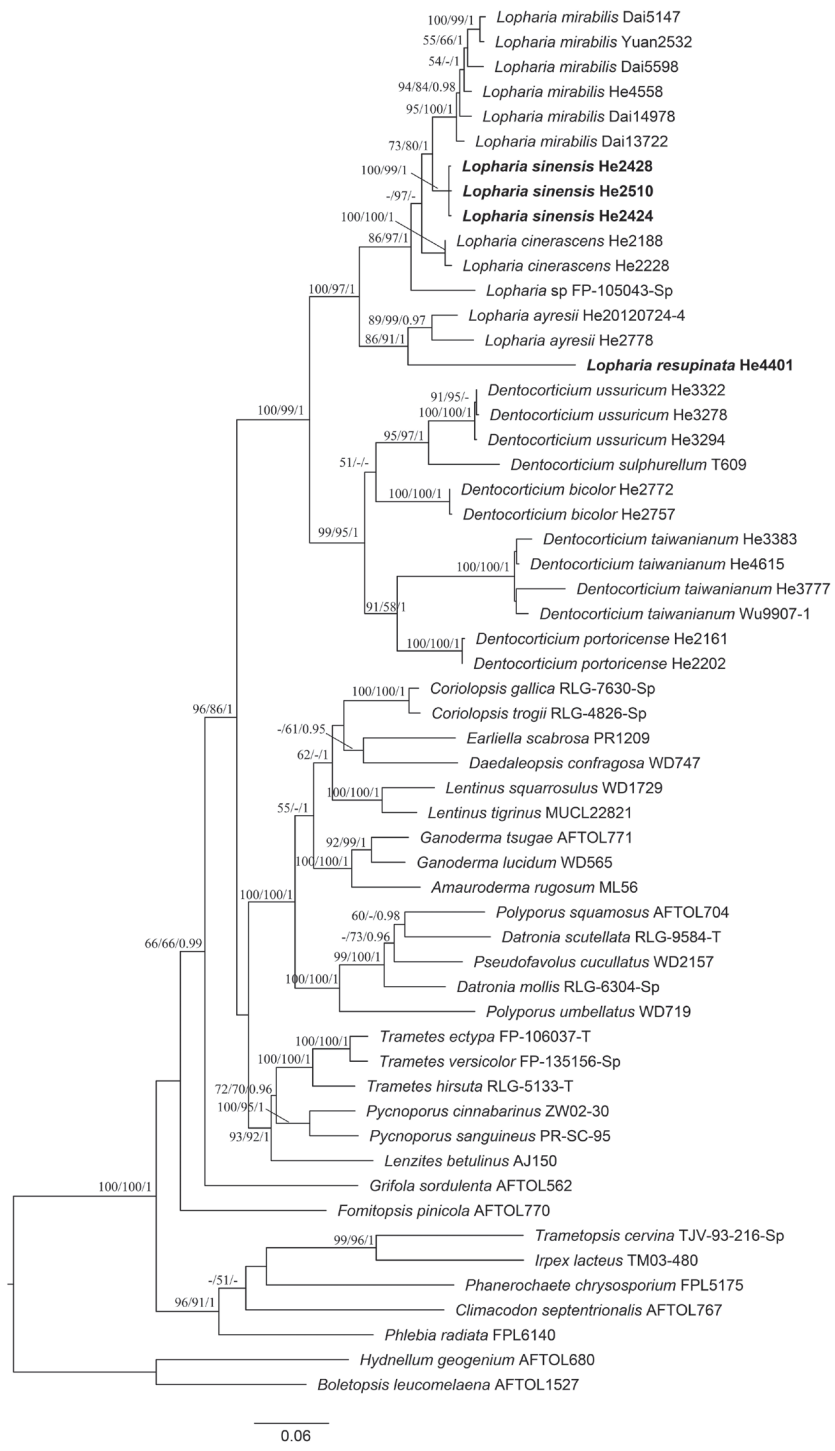
Phylogenetic tree inferred from maximum likelihood analysis of the combined ITS, 28S and *rpb2* sequences of taxa in Polyporales. Branches are labelled with maximum likelihood and maximum parsimony bootstrap values ≥ 50 % and Bayesian posterior probabilities ≥ 0.95.

The ITS-28S-*rpb2* sequences dataset contained 54 ITS, 55 nuc 28S and 40 *rpb2* sequences from 56 samples representing 38 ingroup and 2 outgroup taxa (Table 1). Twenty-three ITS, 25 nuc 28S and 11 *rpb2* sequences were generated for this study (Table 1). The dataset had an aligned length of 2806 characters, of which 836 were parsimony informative. MP analysis yielded four equally parsimonious trees (TL = 5240, CI = 0.323, RI = 0.594, RC = 0.192, HI = 0.677). The best model estimated and applied in the Bayesian analysis was GTR+I+G. MP and BI analyses resulted in almost the same tree topologies as that of ML analysis, which is similar to that of Justo and Hibbett (2011). Only the ML tree is shown in Fig. 1 with maximum likelihood and maximum parsimony bootstrap values ≥ 50 % and BPP ≥ 0.95 labelled along the branches. In the tree, the Dentocorticium clade sensu Justo and Hibbett (2011) was recovered and strongly supported. The five species of *Lopharia* s.s. and FP-105043 (as *Lopharia* sp.) are in a strongly supported lineage with two subclades – (1) *Lopharia sinensis*, *L. mirabilis* and *L. cinerascens* and (2) *L. resupinata* and *L. ayresii*. The *Dentocorticium* species are in a clade with five distinct and well-supported lineages representing the species *D. ussuricum*, *D. sulphurellum*, *D. bicolor*, *D. taiwanianum* and *D. portoricense*.

## Taxonomy of *Lopharia* species

### Lopharia resupinata

Taxon classificationFungiPolyporalesPolyporaceae

S.H. He, S.L. Liu & Y.C. Dai sp. nov.

823071

Figs 2A–B , 3

#### Diagnosis.

Distinguished from other *Lopharia* species by its resupinate basidiocarps, a densely compact texture, a monomitic hyphal system and small basidiospores 7–9(–10) × 4–5 µm.

#### Holotype.

CHINA. Jiangxi Province: Anyuan County, Sanbaishan Forest Park, on fallen angiosperm branch, 15 Aug. 2016, He 4401 (holotype, BJFC 023842!).

#### Etymology.

“*resupinata*” (Lat.) refers to the resupinate basidiocarps.

**Figure 2.**
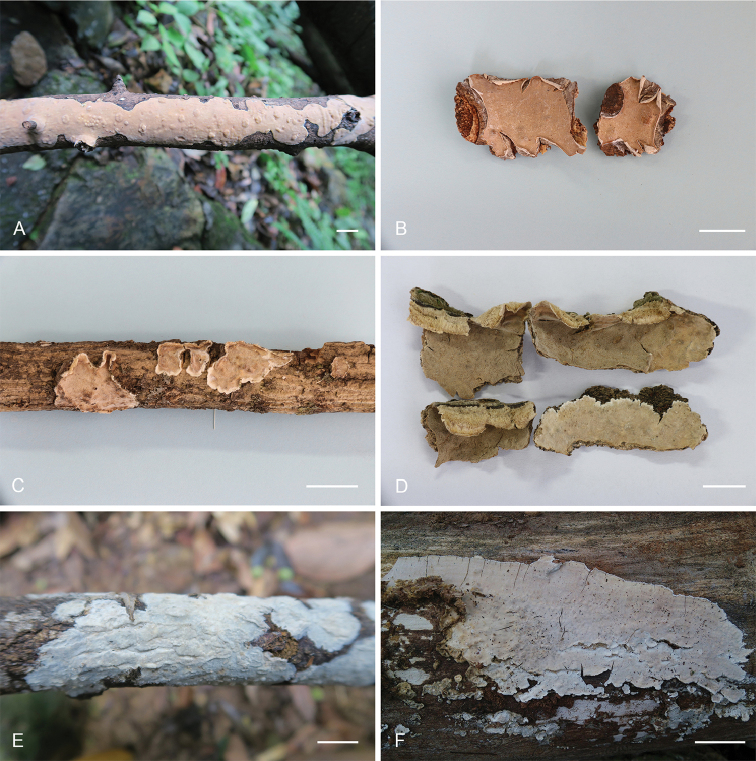
Basidiocarps of *Lopharia* species. **A–B** *L. resupinata* (holotype, He 4401) **C–D** *L. sinensis* (**C** holotype, He 2428 **D** He 2510) **E** *L. ayresii* (He 3884) **F** *L. cinerascens* (He 2228). Scale bars: 1 cm.

#### Fruiting body.

Annual, resupinate, adnate, ceraceous, hygrophanous, not separable from the substrate when fresh, becoming crustaceous, brittle and easily detached from substrate upon drying, first as small patches, later confluent up to 20 cm long, 2.5 cm wide, up to 400 µm thick. Hymenophore smooth, under a lens pilose from projecting cystidia, pale orange (6A3), orange grey (6B2) to greyish-orange (6B3) when fresh, becoming brownish-orange [6C(2–4)] to light brown [6D(4–5)] upon drying, uncracked; margin abrupt, concolorous when fresh, reflexed and incurved upon drying, abhymenial surface white (6A1).

#### Microscopic structures.

Hyphal system monomitic, generative hyphae with clamp connections. Subiculum thin, with numerous small crystals; hyphae hyaline, thin- to slightly thick-walled, moderately septate and branched, interwoven, 2–3.5 µm in diam. Subhymenium thickening, up to 300 µm thick; hyphae hyaline, slightly thick-walled, vertically arranged, densely agglutinated, 2–4 µm in diam. Lamprocystidia abundant, arising from subhymenium, subulate, heavily encrusted with crystals, distinctly thick-walled, embedded in subhymenium or exerted, 80–150 × 10–20 µm. Basidia clavate, with a basal clamp connection and four sterigmata, 50–65 × 8–10 µm; basidioles dominating in hymenium, similar to basidia but smaller. Basidiospores ellipsoid, hyaline, thin-walled, smooth, containing a large guttule, IKI–, CB–, 7–9(–10) × 4–5 µm, L = 7.9 µm, W = 4.4 µm, Q = 1.81 (n = 30/1).

#### Remarks.

*Lopharia resupinata*, like *L. ayresii*, has a resupinate habit, a monomitic hyphal system and a densely compact texture. *Lopharia ayresii* (Fig. 2E), however, has larger basidiospores (11.2 ± 0.7 × 6.4 ± 0.4 µm, from type, Boidin and Gilles 1991). In Fig. 1, *L. resupinata* and *L. ayresii* cluster together. *Lopharia cinerascens* and *L. mirabilis* differ from *L. resupinata* by having effused-reflexed to pileate basidiocarps, a dimitic hyphal system and larger basidiospores (Hjortstam and Ryvarden 1990, Boidin and Gilles 2002). *Lopharia resupinata* has a thickening subhymenium with embedded lamprocystidia, characters that are also found in species of *Phlebiopsis* Jülich.

**Figure 3.**
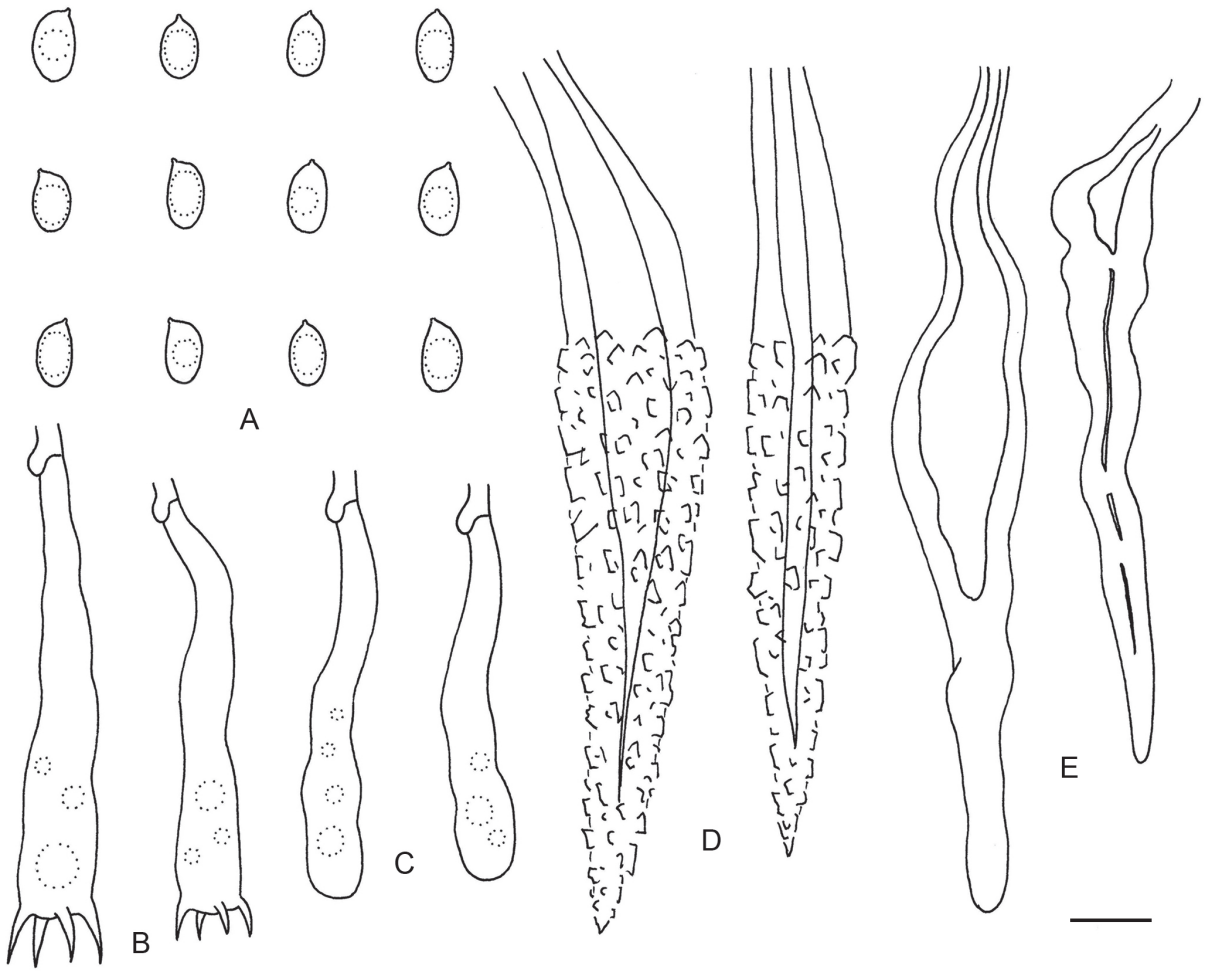
Microscopic structures of *Lopharia resupinata* (drawn from the holotype). **A** Basidiospores **B** Basidia **C** Basidioles **D–E** Lamprocystidia (**D** in cotton blue **E** in KOH).

### Lopharia sinensis

Taxon classificationFungiPolyporalesPolyporaceae

S.H. He, S.L. Liu & Y.C. Dai sp. nov.

823072

Figs 2C–D , 4

#### Diagnosis.

Differs from *L. cinerascens* by its ellipsoid basidiospores and long, projecting cystidia. Known only from northern China.

#### Holotype.

CHINA. Ningxia Autonomous Region: Jingyuan County, Liupanshan Forest Park, on dead angiosperm branch, 4 Aug. 2015, He 2428 (holotype, BJFC 020881!).

#### Etymology.

“*sinensis*” (Lat.) refers to the type locality in China.

#### Fruiting body.

Annual, effused to effused-reflexed, adnate, coriaceous, first as small patches, later confluent, effused part up to 8 cm long, 2.5 cm wide, up to 1 mm thick, pilei projecting up to 1 cm, 3 cm wide. Abhymenial surface tomentose to glabrous, greyish-orange (6B3) to brownish-grey [6D(2–4)]. Hymenophore smooth, greyish-orange (6B3), greyish-brown (6D3) to light brown [6D(4–6)], uncracked; margin thinning out, lighter than hymenophore surface, up to 1.5 mm wide, becoming indistinct and concolorous with age.

**Figure 4.**
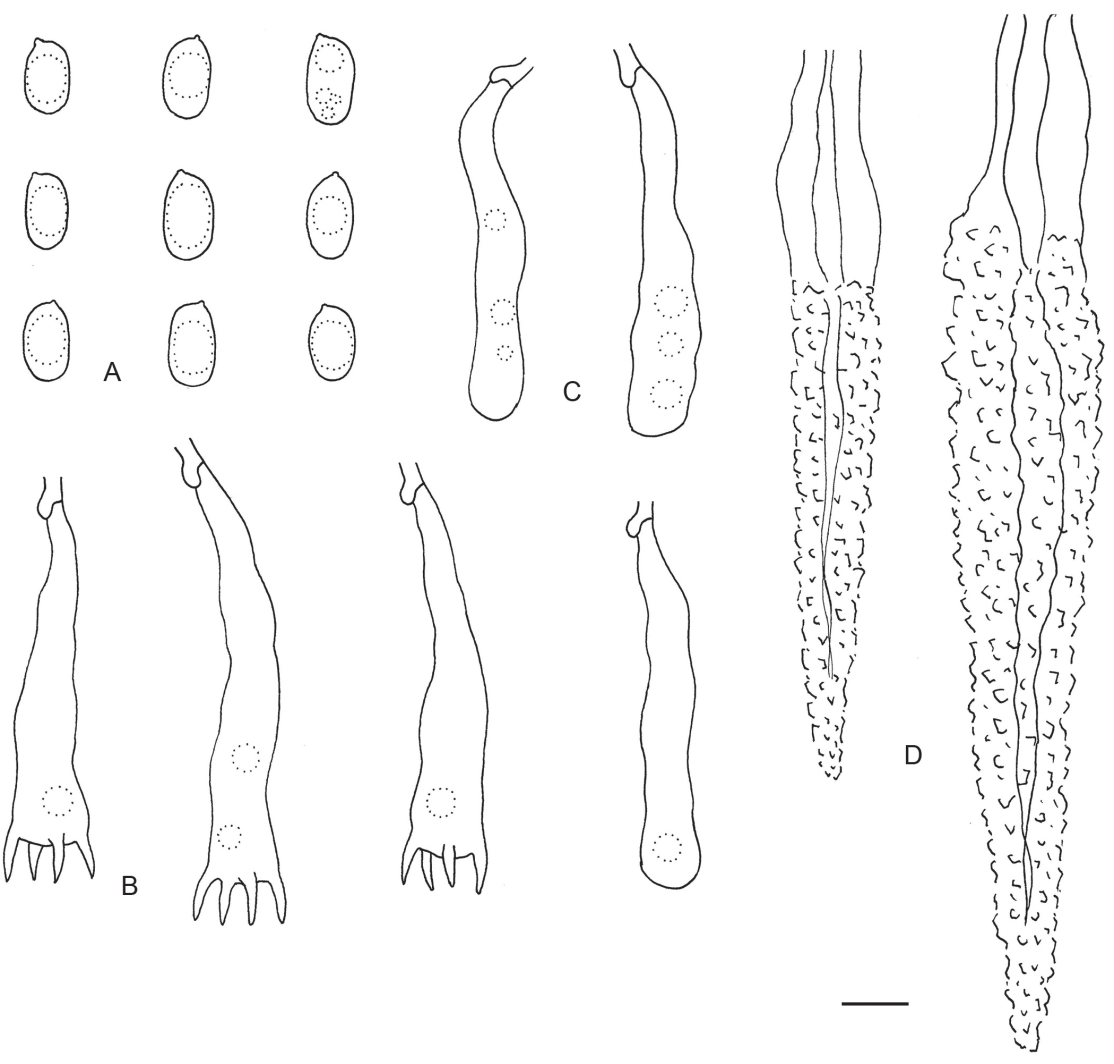
Microscopic structures of *Lopharia sinensis* (drawn from holotype). **A** Basidiospores **B** Basidia **C** Basidioles **D** Lamprocystidia.

#### Microscopic structures.

Hyphal system dimitic, generative hyphae with clamp connections. Cortex and tomentum present. Subiculum well developed, hyphae more or less regularly arranged, interwoven. Skeletal hyphae dominant, thick-walled, pale yellow, unbranched and septate, flexuous, 3–6 µm in diam. Generative hyphae hyaline, thin- to slightly thick-walled, rarely branched and septate, 2–4 µm in diam. Lamprocystidia abundant, large, subulate, distinctly thick-walled, arising from subhymenium, 100–280 × 8–20 µm, projecting up to 200 µm beyond hymenium. Basidia clavate, with a basal clamp and four sterigmata, 45–70 × 9–13 µm; basidioles dominating in hymenium, in shape similar to basidia, but smaller. Basidiospores ellipsoid, hyaline, thin-walled, smooth, containing a large guttule, IKI–, CB–, 11–14 × (6–)6.5–8 µm, L = 12.6 µm, W = 7.1 µm, Q = 1.75–1.79 (n = 60/2).

#### Additional specimens examined.

CHINA. Gansu Province: Pingliang County, Kongtongshan Forest park, on fallen trunk of *Euonymus maackii*, 3 Aug 2015, He 2401 (BJFC 020855); on dead angiosperm branch, 3 Aug 2015, He 2408 (BJFC 020862); Tianshui County, Dangchuan Forest Farm, on construction wood, 8 Aug 2015, He 2510 (BJFC 020963). Hebei Province: Xinglong County, Wulingshan Nature Reserve, on fallen angiosperm branch, 2 Sep 2017, He 5005 (BJFC). Ningxia Autonomous Region: Jingyuan County, Liupanshan Forest Park, on dead angiosperm trunk, 4 Aug 2015, He 2424 (BJFC 020877) & He 2438 (BJFC 020891).

#### Remarks.

*Lopharia sinensis* belongs to the *L. cinerascens* clade (Fig. 1). It differs from *L. mirabilis* by its smooth hymenophore surface and north temperate distribution and from *L. cinerascens* by its ellipsoid basidiospores and long, projecting cystidia (Hjortstam and Ryvarden 1990, Dai 2002). *Lopharia pseudocinerascens* from Africa also belongs to the *L. cinerascens* group and can be distinguished from *L. sinensis* by narrower basidiospores (8–14 × 4.5–6.5 µm, Boidin and Gilles 2002).

**Figure 5.**
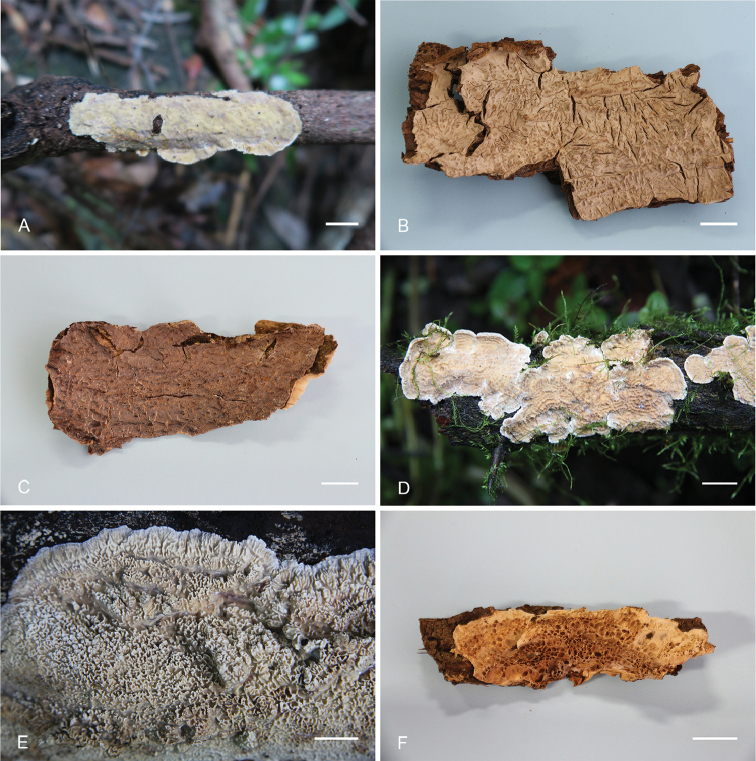
Basidiocarps of *Lopharia mirabilis*. **A** He 4558 **B** Dai 15094 **C** Dai 14978 **D** He 20120923-7 **E** He 1657 **F** Cui 9330.

Six species of *Lopharia*, *L. ayresii*, *L. cinerascens*, *L. resupinata*, *L. mirabilis*, *L. sinensis* and *Lopharia* sp. (FP-105043) are included in a fully supported monophyletic clade (Fig. 1). They all develop the large encrusted cystidia, the large basidia (> 50 µm long) and the relatively large basidiospores (> 8 µm long and 4 µm wide) that characterise the genus. *Lopharia mirabilis*, the generic type, is a tropical species possessing a tuberculate, odontoid, irpicoid to semiporoid hymenophore (Hjortstam and Ryvarden 1990, Dai 2002). The authors’ phylogenetic analyses show that collections from temperate to tropical areas in China, with smooth to semiporoid hymenophores, cluster together, thus extending the geographical range and hymenophore variability for *L. mirabilis* (Figs 1, 5). Thus, specimens from Taiwan, previously identified as *L. cinerascens* (Boidin and Gilles 2002, Wu 2010) because of their smooth hymenophore, are in fact *L. mirabilis*.

*Lopharia cinerascens* is a cosmopolitan species in temperate to subtropical areas (Hjortstam and Ryvarden 1990, Boidin and Gilles 2002). These phylogenetic analyses suggest that it is a species complex (Fig. 1). Two specimens (He 2188 and He 2228, Fig. 2F) from Wisconsin in northern United States are probably *L. cinerascens* s.s. for it is near the type locality of Pennsylvania. They are phylogenetically distinct from FP-105043 (listed as *L. cinerascens* in Justo and Hibbett, 2011) which was collected in Mississippi, southern United States.

*Lopharia ayresii* nests within the *Lopharia* clade and forms with *L. resupinata* a strongly supported lineage sister to the *L. mirabilis* group (Fig. 1). These two species have resupinate basidiocarps, a monomitic hyphal system, a thin to indistinct subiculum and a thickened subhymenium. Otherwise, they fit well with other *Lopharia* species in developing large basidia and basidiospores and encrusted cystidia. The addition of these species requires that the genus description of *Lopharia* be modified to include monomitic taxa.

It is still premature to make a conclusion about the distribution of *Lopharia* species with present data. Three species, *L. pseudocinerascens*, *L. sinensis* and *L. resupinata*, have been found from the type localities only (Boidin and Gilles 2002, present study). *Lopharia mirabilis* is reported from tropical Africa to temperate to tropical East Asia (Hjortstam and Ryvarden 1990, present study). *Lopharia ayresii* seems to be pantropical and is reported from Mauritius, Réunion (Boidin and Gilles 1991), southern China (Wu 2008), Taiwan (Wu 2010), Okinawa (Maekawa et al. 2003) and South America (Hjortstam et al. 2005, Hjortstam and Ryvarden 2008).

### Lopharia

Taxon classificationFungiPolyporalesPolyporaceae

Kalchbr. & MacOwan, Grevillea 10: 58, 1881, emended

#### Note.

Basidiocarps annual, effused, effused-reflexed or pileate, crustaceous, coriaceous or corky. Pilei tomentose to glabrous. Hymenophore surface smooth, tuberculate, odontoid, irpicoid to semiporoid, cream, greyish-brown to light brown. Hyphal system monomitic or dimitic; generative hyphae with clamp connections. Lamprocystidia metuloid, large, subulate, hyaline, distinctly thick-walled. Dendrohyphidia absent, simple hyphidia hyphoid, thin-walled, hyaline. Basidia clavate with 4 sterigmata, large (> 50 µm long). Basidiospores ellipsoid to cylindrical, hyaline, thin-walled, smooth, negative in Melzer’s reagent, acyanophilous.

#### Type species.

*Lopharia mirabilis* (Berk. & Broome) Pat., *Bulletin de la Société Mycologique de France* 11: 14, 1895.

#### Key to species of *Lopharia* s.s.

**Table:** 

1	Hymenophore tuberculate, odontoid, irpicoid to subporoid	***L. mirabilis***
–	Hymenophore smooth or slightly tuberculate	**2**
2	Basidiocarps effused-reflexed to pileate; hyphal system dimitic	**3**
–	Basidiocarps resupinate; hyphal system monomitic	**6**
3	Basidiospores 4.5–6.5 µm wide; reported from Africa	***L. pseudocinerascens***
–	Basidiospores 6.5–8 µm wide	**4**
4	From Taiwan	***L. mirabilis***
–	From elsewhere	**5**
5	Cystidia projecting up to 70 µm; basidiospores Q value > 1.9; from northern United States	***L. cinerascens***
–	Cystidia projecting up to 200 µm; basidiospores Q value < 1.9; from northern China	***L. sinensis***
6	Basidiospores > 10 µm long	***L. ayresii***
–	Basidiospores < 10 µm long	***L. resupinata***

### List of names in *Lopharia* and their current taxonomic status

The list by species epithet is obtained from Index Fungorum (http://www.indexfungorum.org, 25 Sep. 2017). If a name is accepted, a direct statement is made with supporting evidence cited. Note that Miettinen et al. (2017: 26) consider *Hjortstamia* Boidin & Gilles to be a synonym of *Phlebiopsis* based on molecular and morphological criteria. Hjortstam and Ryvarden (1990) compiled the first nomenclature of *Lopharia* species.

***abietina*** (Pers.) Z.S. Bi & G.Y. Zheng, [Macrofungus flora of the mountainous district of North Guangdong]: 62 (1990). Accepted as ***Veluticeps abietina*** (Pers.) Hjortstam & Tellería. Supported by ITS (Yang et al. 2016) and multi-gene phylogenetic analyses (Garcia-Sandoval et al. 2011).

***albida*** Rick, *Brotéria, Ci. Nat.* 7: 13 (1938). An unidentifiable species of ***Hyphodontia*** as reported by Hjortstam and Ryvarden (1990: 59) and Baltazar et al. (2016: 119) for the type is sterile.

***americana*** Rick, *Egatea* 13: 435 (1928). Hjortstam and Ryvarden (1990: 59) reported that the type is lost.

***amethystea*** (Hjortstam & Ryvarden) A.L. Welden, *Flora Neotropica Monograph* 106: 70 (2010). = ***Hjortstamia amethystea*** (Hjortstam & Ryvarden) Boidin & Gilles. Hjortstam and Ryvarden (1990: 29) observed that the species is close to Porostereum (Phlebiopsis) crassum (Lév.) Hjortstam & Ryvarden.

***areolata*** G. Cunn., Bull. *New Zealand Dept. Sci. Industr. Res.* 145: 331 (1963). = ***Phanerochaete areolata*** (G.H. Cunn.) Hjortstam & Ryvarden. Welden (1975: 547) noted that the type was related to the genus *Phanerochaete*. Hjortstam and Ryvarden (1990: 59) also examined the type and pointed out similarities to *Phanerochaete hiulca* (Burt) Welden.

***ayresii*** (Berk. ex Cooke) Hjortstam, *Mycotaxon* 54: 188 (1995). Accepted in ***Lopharia*** and supported by phylogenetic analyses (fig. 1 herein). The type (Kew 35450, Mauritius, P.B. Ayres) was examined.

***bambusae*** Rick, *Iheringia* 7: 199 (1960). Accepted as a synonym of ***Fomitiporia bambusarum*** (Rick) Campos-Santana & Decock. Hjortstam and Ryvarden (1990: 59) and Baltazar et al. (2016: 119) examined the type and agreed that it belongs to the Phellinus (Fomitiporia) punctatus species complex.

***cheesmanii*** (Wakef.) G. Cunn., Bull. *New Zealand Dept. Sci. Industr. Res.* 145: 195 (1963). Accepted as a synonym of ***Laurilia sulcata*** (Burt) Pouzar as proposed by Hjortstam and Ryvarden (1990: 59) who examined the type at Kew. In addition, Boidin (1969: 190) observed finely echinulate, amyloid basidiospores in the type specimen.

***cinerascens*** (Schwein.) G. Cunn., *Trans. Roy. Soc. New Zealand* 83: 622 (1956). Accepted in ***Lopharia*** and supported by phylogenetic analyses (fig. 1 herein).

***crassa*** (Lév.) Boidin, *Bull. Trimestriel Soc. Mycol. France* 74: 479 (1959). Accepted as ***Phlebiopsis crassa*** (Lév.) Floudas & Hibbett and supported by multi-gene phylogenetic analyses; see (Floudas and Hibbett 2015: figs 1, 3) and (Miettinen et al. 2016: fig. 2 part 2).

***cystidiosa*** (Rehill & B.K. Bakshi) Boidin, *Rev. Mycol.* (*Paris*) 34: 191 (1969). = ***Porostereum cystidiosum*** (Rehill & B.K. Bakshi) Hjortstam & Ryvarden.

***dregeana*** (Berk.) P.H.B Talbot, *Bothalia* 6: 57 (1951). = ***Australohydnum dregeanum*** (Berk.) Hjortstam & Ryvarden.

***fulva*** (Lév.) Boidin, *Bull. Mens. Soc. Linn. Lyon* 28: 213 (1959). Accepted as ***Porostereum fulvum*** (Lév.) Boidin & Gilles. Although considered a synonym of *P. spadiceum* by Hjortstam and Ryvarden (1990: 61), Boidin and Gilles (2002: 109) showed by crossing experiments and differences in basidiospore shape and size that *P. fulvum* was distinct from *P. spadiceum*. Welden (1975) also noted basidiospore size differences. In addition, they have distinct distributions — *P. fulvum* is reported from Africa, Reunion, India, Pakistan, Nepal, Philippines, Australia, New Zealand and Siberia, whereas *P. spadiceum* is known from Europe, Armenia and Morocco (Boidin and Gilles 2002, Talbot 1954, Welden 1975).

***heterospora*** (Burt) D.A. Reid, *Rev. Mycol.* (*Paris*) 33: 251 (1969). Accepted as a synonym of ***Dendrophora albobadia*** (Schwein.) Chamuris. Welden (1975: 547), Boidin and Lanquetin (1977: 120) and Chamuris (1987) examined the type specimen, Matthews 27 and agreed that it is conspecific with ***D. albobadia***.

***involuta*** (Klotzsch) G. Cunn., *Bull. New Zealand Dept. Sci. Industr. Res.* 145: 194 (1963). = ***Podoscyha involuta*** (Klotzsch) Imazeki. In a phylogenetic study of stipitate stereoid fungi, Sjökvist et al. (2012) showed that *Podoscypha* was paraphyletic with *P. involuta* and two other species in a lineage separate from the larger group of *Podoscypha* species.

***javanica*** Henn. & E. Nyman, *Monsunia* 1: 144 (1900) [1899]. A possible synonym of *L. mirabilis* (Talbot 1954: 342; Boidin 1959: 207) or *L. cinerascens* (Welden 1975: 536). A portion of the type may be at NY (no. 00775916).

***lilacina*** (Berk. & Broome) A.L. Welden, *Flora Neotropica Monograph* 106: 71 (2010). = ***Porostereum lilacinum*** (Berk. & Broome) Hjortstam & Ryvarden.

***lirellosa*** Kalchbr. & MacOwan, in Kalchbrenner, *Grevillea* 10 (54): 58 (1881). Accepted as a synonym of ***L. mirabilis*** as proposed by Talbot (1951: 56; 1954: 340). Hjortstam and Ryvarden (1990: 62) and Boidin and Gilles (2002: 94) follow Talbot’s synonymy.

***mexicana*** A.L. Welden, *Tulane Stud. Zool. Bot.* 17: 19 (1971). = ***Hjortstamia mexicana*** (A.L. Welden) Boidin & Gilles.

***mirabilis*** (Berk. & Broome) Pat., *Bull. Soc. Mycol. France* 11: 14 (1895). Type species of ***Lopharia***.

***novae-granata*** A.L. Welden, *Mycologia* 67: 540 (1975). = ***Hjortstamia novae-granata*** (Welden) Hjortstam & Ryvarden.

***ochracea*** G. Cunn., *Bull. New Zealand Dept. Sci. Industr. Res.* 145: 196 (1963). Accepted as ***Amylostereum areolatum*** (Fr.) Boidin based on basidiospore size (Thomsen, 1998) and its occurrence in New Zealand (Talbot 1964, Gaut 1969). Boidin and Lanquetin (1984) identified two paratype specimens as a species of *Amylostereum*. Hjortstam and Ryvarden (1990: 62) reported that the type specimen was morphologically indistinguishable from *A. chailletii* (Fr.) Boidin.

***papyracea*** (Bres.) D.A. Reid, *Kew Bull.* 12: 131 (1957). Accepted as ***Phlebiopsis friesii*** (Lév.) Spirin & Miettinen. Originally published as *L. papyracea* (Jungh.) D.A. Reid. *Lloydella papyracea* Bres. 1910 is the replacement name for *Thelephora papyracea* Jungh. which is a later homonym of *T. papyracea* Schrader ex J.F. Gmelin 1792.

***papyrina*** (Mont.) Boidin, *Bull. Mens. Soc. Linn. Lyon* 28: 210 (1959). Accepted as ***Phlebiopsis papyrina*** (Mont.) Miettinen & Spirin.

***perplexa*** D.A. Reid, *Kew Bull.* 17: 297 (1963). = ***Hjortstamia perplexum*** (D.A. Reid) Boidin & Gilles.

***phellodendri*** (Pilát) Boidin, *Bull. Mens. Soc. Linn. Lyon* 28: 207 (1959). = ***Porostereum phellodendri*** Pilát, type of *Porostereum*. A possible synonym of *P. fulva* (Boidin and Gilles, 2002: 108) or *P. spadiceum* (Hjortstam & Ryvarden, 1990: 62). See discussion under *L. fulva*.

***pilosiuscula*** (Hjortstam & Ryvarden) A.L. Welden, *Fl. Neotrop. Monogr.* 106: 73 (2010). Placement is uncertain for it is not typical of *Porostereum* (Hjortstam and Ryvarden 1990: 49) nor of *Lopharia* s.s. (Welden 2010: 73).

***pseudocinerascens*** Boidin & Gilles, *Bull. Trimestriel Soc. Mycol. France* 118: 96 (2002). Accepted in ***Lopharia***.

***rhodocarpa*** (Rehill & B.K. Bakshi) S.S. Rattan, *Biblioth. Mycol.* 60: 172 (1977). Accepted as ***Peniophora rhodocarpa*** Rehill & B.K. Bakshi. The authors follow Hjortstam & Ryvarden (1990: 62) who examined the isotype at Kew.

***rimosissima*** Rick in Rambo, Iheringia, *Ser. Bot.* 7: 199 (1960). The protologue does not provide enough information to identify this species but it may be a *Xylodon* species. A line after the protologue states that it appears to be identical to *Odontia rimosissima* Peck [= *Xylodon rimosissimus* (Peck) Hjortstam & Ryvarden].

***rimosissima*** (Berk. & M.A. Curtis) A.L. Welden, *Mycologia* 67: 544 (1975). = ***Hjortstamia rimosissima*** Boidin & Gilles. Known only from the type from Nicaragua collected on dead cane. Although the type lacks basidiospores, it is otherwise similar to *P. crassa* (Burt 1925: 342; Welden 1975: 544, 2010: 73).

***rugulosa*** (Berk. & M.A. Curtis) Hjortstam, *Mycotaxon* 54: 188. 1995. Of uncertain generic disposition because of conflicting observations of the type specimen (Ginns 1971: 230, Hjortstam 1990: 420, Ryvarden 2010: 115).

***sharpiana*** A.L. Welden, *Tulane Stud. Zool. Bot.* 17: 18 (1971). = ***Porostereum sharpianum*** (A.L. Welden) Hjortstam & Ryvarden. Hjortstam and Ryvarden (1990: 51) made the transfer after examining the type specimen. Welden (2010: 74), however, believed it is better placed in *Lopharia* s.s.

***spadicea*** (Pers.) Boidin, *Bull. Mens. Soc. Linn. Lyon* 28: 211 (1959). Accepted as ***Porostereum spadiceum*** (Pers.) Hjortstam & Ryvarden. See *L. fulva* for additional information.

***umbrinoalutacea*** (Wakef.) A.L. Welden, *Mycologia* 67: 546 (1975). Accepted as ***Porostereum umbrinoalutacea*** (Wakef.) Hjortstam & Ryvarden. Hjortstam and Ryvarden (1990: 63) made the transfer to *Porostereum* after examining the type specimen. Welden (1975: 539) noted that *P. umbrinoalutacea* was closely related to *P. fulvum* and *P. spadiceum*.

***vinosa*** (Berk.) G. Cunn., *Trans. Roy. Soc. New Zealand* 83: 625 (1956). Accepted as a synonym of ***Phlebiopsis crassa***. Lentz (1955: 20), (Cunningham 1956: 624, fig. 2) and Hjortstam and Ryvarden (1990: 63) examined the type of *Corticium vinosum* Berk. They all agree that *C. vinosum* is conspecific with *Thelephora crassa* Lév. Note that some authors have mistakenly used *Thelephora vinosa* Berk. instead of *Corticium vinosum* Berk. as the proper basionym; see May et al. (2003: 295) for a summary.

### Taxonomy of *Dentocorticium*, *Dendrodontia* and *Fuscocerrena* species

**Figure 6.**
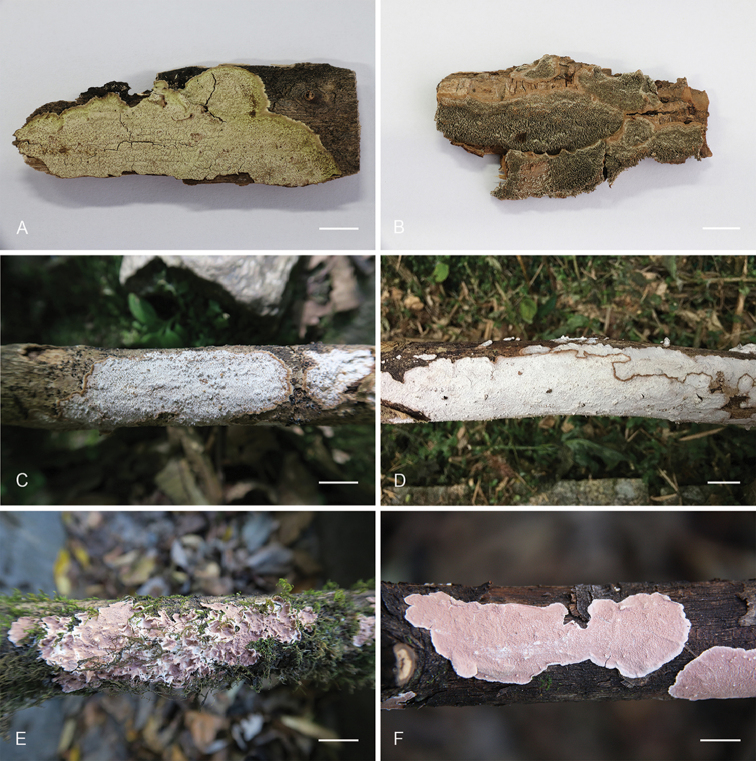
Basidiocarps of *Dentocorticium* species. **A** *D. bicolor* (He 2757) **B** *D. portoricense* (He 2161) **C–D** *D. taiwanianum* (**C** He 3383 **D** He 4635) **E–F** *D. ussuricum* (**E** He 3278 **F** He 3294). Scale bars: 1 cm.

*Dendrodontia bicolor* (generic type, Fig. 6A), *Fuscocerrena portoricensis* (generic type, Fig. 6B), *Dentocorticium sulphurellum*, *Dentocorticium taiwanianum* (Fig. 6C–D) and *Dentocorticium ussuricum* (Parmasto) M.J. Larsen & Gilb. (generic type, Fig. 6E–F) cluster in a strongly supported clade (Fig. 1). The phylogenetic analyses demonstrate that the three genera are closely related and support merging the genera together. Amongst the three generic names, *Dentocorticium* (1974) has priority over *Dendrodontia* (1980) and *Fuscocerrena* (1982). Thus, the latter two genera are treated as synonyms of *Dentocorticium* and four new combinations are proposed. An expanded and more inclusive generic circumscription of *Dentocorticium* is presented below.

### Dentocorticium

Taxon classificationFungiPolyporalesPolyporaceae

(Parmasto) M.J. Larsen & Gilb., Norwegian Journal of Botany 21: 225, 1974, emended

Laeticorticium sect. Dentocorticium Parmasto, Conspectus Systematis Corticiacearum: 151, 1968; Dendrodontia Hjortstam & Ryvarden, *Mycotaxon* 10: 273, 1980; Fuscocerrena Ryvarden, *Transactions of the British Mycological Society* 79: 279, 1982.

#### Note.

Basidiocarps annual, effused, effused-reflexed or pileate, membranous, coriaceous or soft corky. Hymenophore surface odontoid, tuberculate, spinose, poroid, daedaleoid, sometimes developing irregular ridges or hyphal pegs. Hyphal system dimitic or trimitic; generative hyphae with clamp connections, brown skeletal hyphae in subiculum, spine trama and hyphal pegs, microbinding hyphae may be present in subiculum or substrate. Dendrohyphidia present. Cylindrical to subfusiform cystidia may be present. Basidia clavate with 4 sterigmata. Basidiospores ellipsoid to cylindrical, hyaline, thin-walled, smooth, negative in Melzer’s reagent, acyanophilous.

#### Type species.

*Laeticorticium ussuricum* Parmasto, *Eesti NSV Teaduste Akadeemia Toimetised* 14: 229, 1965.

#### Key to species of *Dentocorticium*

**Table:** 

1	With hyphal peg	**2**
–	Without hyphal peg	**3**
2	Sterile margin distinct and brown; hyphal pegs 4–5 per mm; subiculum brown	***D. taiwanianum***
–	Sterile margin indistinct; hyphal pegs > 5 per mm; subiculum grey	***D. hyphopaxillosum***
3	Hymenophore poroid or with ridges, hydnoid to spinose, from North and South America	***D. portoricense***
–	Hymenophore smooth, tuberculate, odontoid, rarely spinose	**4**
4	Hymenial surface white to yellow, basidiospores 7–9.5 × 2.5–3 µm long, reported from North America	***D. sulphurellum***
–	Hymenial surface cream, brown to violaceous, basidiospores 5–7 × 2.2–2.5 µm long, reported from East Asia	***D. ussuricum***
–	Hymenial surface cream, yellow or brown, basidiospores 8–9 × 3–4 µm long, reported from southern Africa, Australia, East Asia, North and South America	***D. bicolor***

### Dentocorticium bicolor

Taxon classificationFungiPolyporalesPolyporaceae

(P.H.B. Talbot) Nakasone & S.H. He comb. nov.

823073

Fig. 6A

Dendrodontia bicolor (P.H.B. Talbot) Hjortstam & Ryvarden, *Mycotaxon* 10: 273, 1980.

#### Basionym.

*Grandinia bicolor* P.H.B. Talbot, *Bothalia* 4: 947, 1948.

#### Type specimen examined.

South Africa: Natal Province: Pietermaritzburg District, Town bush valley, on dead wood, Aug. 1934, W.G. Rump 100, UDA Herb. No. 27756 [K, K(M)15722, holotype].

#### Other specimens examined.

China. Anhui Province: Qimen County, Guniujiang Nature Reserve, on fallen angiosperm branch, 8 Aug 2013, He 1722 (BJFC 016189, CFMR). Yunnan Province: Yongde County, Daxueshan Nature Reserve, on dead *Juglans* branch, 28 Aug 2015 He 2757 (BJFC 021195, CFMR) & He 2772 (BJFC 021210, CFMR). Zhejiang Province: Lin’an County, Tianmushan Nature Reserve, on dead angiosperm branch, 6 Aug 2013, He 1691 (BJFC 016158, CFMR). South Africa, Natal Province, Pietermaritzburg District, Town bush, on (corticated) indigenous wood, Oct 1934, W.G. Rump 215, herb.no. 28291, W.G. Rump 217, herb no. 28292, W.G. Rump 270 herb. No. 28502 (PREM).

#### Remarks.

See Hjortstam and Ryvarden (1980) for a description and illustration of this species. The authors were unable to obtain sequences of *Dentocorticium bicolor* from the type locality in South Africa. Maekawa (1994) reported *D. sulphurellum* from Japan; however, the Japanese specimens may be *D. bicolor*, for *D. sulphurellum* appears to be restricted to North America.

### Dentocorticium hyphopaxillosum

Taxon classificationFungiPolyporalesPolyporaceae

(M.J. Li & H.S. Yuan) Nakasone & S.H. He comb. nov.

823080

#### Basionym.

*Dendrodontia hyphopaxillosa* M.J. Li & H.S. Yuan, *Phytotaxa* 156: 183, 2014.

#### Type specimen examined.

China. Guangxi Autonomous Region: Shangsi County, Shiwandashan Forest Park, on fallen angiosperm branch, 24 Jul 2012, Yuan 6269 (CFMR, isotype).

#### Remarks.

Although not included in phylogenetic analyses, this combination is made based on morphological evidence. See Li and Yuan (2014) for description and illustration.

### Dentocorticium portoricense

Taxon classificationFungiPolyporalesPolyporaceae

(Spreng. ex Fr.) Nakasone & S.H. He comb. nov.

823074

Fig. 6B

Fuscocerrena portoricensis (Spreng. ex Fr.) Ryvarden, *Transactions of the British Mycological Society* 79: 280, 1982.

#### Basionym.

*Polyporus portoricensis* Spreng. ex Fr., *Elenchus Fungorum* 1: 115, 1828.

#### Specimens examined.

Costa Rica. San José Province: Jardin, on hardwood, 9 Aug 1963, J.L. Lowe 13402 (CFMR). Uruguay. Depto. Tacuarembó, Ext. Paso Baltasar, on *Eucalyptus globulus*, 11 Nov 2001, L. Bettucci and S. Lupo, MVHC 5038 (CFMR). USA. Florida: Alachua County, Devil’s Millhopper, on *Magnolia* sp., 18 July 1972, H.H. Burdsall, Jr., HHB 19632 (CFMR). Tennessee: Cocke County, Cosby Nature Trail, on *Liriodendron tulipifera* log, 2 Aug 2010, H.H. Burdsall, Jr., HHB 6651 (CFMR). Wisconsin: Dane County, Madison, Picnic Point, on dead angiosperm tree, 7 Oct 2014, He 2161 (BJFC 018806, CFMR); 11 Oct 2014, He 2202 (BJFC 018832, CFMR).

#### Remarks.

*Dentocorticium portoricense* is easily recognised by its poroid, hydnoid to spinose, dark brown hymenophore and greenish-yellow hymenial surface. Phylogenetically, it is closely related to *D. taiwanianum* (Fig. 1). See Ryvarden (1982) for description and drawing of this species with synonymy.

### Dentocorticium taiwanianum

Taxon classificationFungiPolyporalesPolyporaceae

(H.C. Wang & Sheng H. Wu) Nakasone & S.H. He comb. nov.

823075

Fig. 6C–D

#### Basionym.

*Dendrodontia taiwaniana* H.C. Wang & Sheng H. Wu, *Mycologia* 102: 1153, 2010.

#### Type specimen examined.

Taiwan: Nantou County, Hsitou, alt. 1000 m, on (corticate) branch of angiosperm, 3 Jul. 1999, *S.H. Wu 9907-1*, F10258 (TNM, holotype).

#### Other specimens examined.

China. Guizhou Province: Libo County, Maolan Nature Reserve, on dead angiosperm branch, 14 Jun 2016, He 3777 (BJFC 022276). Hainan Province: Wuzhishan County, Wuzhishan Nature Reserve, on dead angiosperm branch, 10 Jun 2016, He 3927 (BJFC 022429). Taiwan: Nantou County, Nandongyan Mountains, on fallen angiosperm trunk, 7 Dec 2016, He 4615 (BJFC 024057); Xitou, on dead angiosperm branch, 11 Dec 2016, He 4635 (BJFC 024078) & He 4639 (BJFC 024082). Yunnan Province: Baoshan County, Baihualing, on fallen angiosperm branch, 30 Nov 2015, He 3383 (BJFC 021778).

#### Remarks.

This is a common species in tropical China. See Wang et al. (2010) for a description and illustration of this species.

### List of names in *Dentocorticium* and their current taxonomic status

The list by species epithet is obtained from Index Fungorum (http://www.indexfungorum.org, 25 Sep. 2017). If a name is accepted, a direct statement is made with supporting evidence cited.

***blastanos*** Boidin & Gilles, *Cryptog. Mycol.* 19: 193 (1998). Accepted as ***Neocampanella blastanos*** (Boidin & Gilles) Nakasone, Hibbett & Goranova and supported by molecular data (Nakasone et al. 2009: fig. 1).

***brasiliense*** M.J. Larsen & Gilb., *Norweg. J. Bot.* 24: 117 (1977). Accepted as ***Punctularia subhepatica*** (Berk.) Hjortstam. The isotype at CFMR (Brazil, Rio Grande du Sol, ad ligna angiosperma, 1936, Rick) was examined. It has rare basidiospores (6.5–8.7 × 3.2–3.7 µm) and characteristic knobby dendrohyphidia that are brown in the upper portion and hyaline at the base. The holotype at FH is apparently lost.

***expallens*** (Bres.) Domański, Mala Flora Grzybów. Tom I: Basidiomycetes (Podstawczaki), Aphyllophorales (Bezblaszkowce). Corticiaeae, *Acanthobasidium* – *Irpicodon* 5: 248 (1988). = ***Crustomyces expallens*** (Bres.) Hjortstam. In addition to *Corticium*, *Dentocorticium*, and *Crustomyces*, this species has been transferred to *Phlebia* and *Laeticorticium*, but none of these generic placements is satisfactory.

***irregulare*** Ryvarden, *Bull. Jardin Bot. Natl. Belg.* 48: 84 (1978). Accepted as a synonym of ***Diplomitoporus daedaleiformis*** (Henn.) Ryvarden. The holotype of *D. irregulare* (JR 4316, GENT) and isotype of *Poria daedaleiformis* (US0239243, BPI) were examined. Basidiospores of *D. irregulare* were narrower [(2.8–) 3–3.5 µm] than reported by Ryvarden (1978) and similar to those of *D. daedaleiformis* (Ryvarden 2012: 16). Also in *D. irregulare*, skeletal hyphae were observed in the ridges and spines and obclavate, subfusiform cystidioles (11.5–21 × 4–5.5 µm) in the hymenium; these were not described earlier. Cystidioles were also observed in the isotype of *P. daedaleiformis* but no basidiospores. Both species develop elongated pores and ridges, clamped generative, dendrohyphidia and occur in the same geographical area in Africa.

***nephrolepidis*** Boidin & Gilles, *Cryptog. Mycol.* 19: 193 (1998). Accepted as a synonym of ***L. cyathae*** (S. Ito & S. Imai) Hjortstam & Ryvarden as determined by Nakasone (2005) who examined the holotype.

***pilatii*** (Parmasto) Duhem & H. Michel, *Cryptog. Mycol.* 30: 165 (2009). Accepted as ***Phlebiopsis pilatii*** (Parmasto) Spirin & Miettinen based on ITS and 28S sequences analyses (Miettinen et al. 2016: fig. 2 part 2). However, ***P. pilatii*** differs from other ***Phlebiopsis*** species in the absence of lamprocystidia and presence of dendrohyphidia and microbinding hyphae (Duhem and Michel 2009: figs 7–17).

***sasae*** (Boidin, Cand. & Gilles) Boidin, Lanq. & Duhem, *Bulletin de la Société Mycologique de France* 112: 116 (1996). Accepted as ***Leptocorticium sasae*** (Boidin, Cand. & Gilles) Nakasone based on morphological criteria (Nakasone 2005).

***sinapicolor*** Boidin, Gilles & Duhem, *Cryptog. Mycol.* 19: 194 (1998). A poorly studied species. Duhem and Michel (2009: 171) cite morphological similarities between this species and ***P. pilatii***.

***sulphurellum*** (Peck) M.J. Larsen & Gilb., *Norweg. J. Bot.* 21: 226 (1974). Accepted in ***Dentocorticium*** as inferred from multi-gene sequences (fig. 1 herein) and morphology.

***ussuricum*** (Parmasto) M.J. Larsen & Gilb., *Norweg. J. Bot.* 21: 226 (1974). This is the generic type of ***Dentocorticium***.

***utribasidiatum*** Boidin & Gilles, *Cryptog. Mycol.* 19: 196 (1998). Accepted as ***Leptocorticium utribasidiatum*** (Boidin & Gilles) Nakasone based on morphological features and examination of the holotype (Nakasone 2005).

## Supplementary Material

XML Treatment for Lopharia resupinata

XML Treatment for Lopharia sinensis

XML Treatment for Lopharia

XML Treatment for Dentocorticium

XML Treatment for Dentocorticium bicolor

XML Treatment for Dentocorticium hyphopaxillosum

XML Treatment for Dentocorticium portoricense

XML Treatment for Dentocorticium taiwanianum
